# The Development and Effectiveness of a Web-Based Emergency Management Educational Program for Long-Term Care Facility Interprofessional Practitioners

**DOI:** 10.3390/ijerph182312671

**Published:** 2021-12-01

**Authors:** Young-Rim Choi, Dai-Young Kwon, Sung-Ok Chang

**Affiliations:** 1 Institute of Nursing Research, College of Nursing, Korea University, Seoul 02841, Korea; cyr0906@korea.ac.kr; 2 Gifted Education Center, Korea University, Seoul 02841, Korea; dykwon.edu@gmail.com; 3 BK21 FOUR R&E Center for Learning Health Systems, College of Nursing, Korea University, Seoul 02841, Korea

**Keywords:** long-term care, emergencies, interprofessional practitioners, shared mental model, web-based educational program

## Abstract

Long-term care facility (LTCF) interprofessional practitioners who care for residents at high risk of emergencies due to old age, frailty, and complex diseases must be able to manage such emergencies collaboratively. A shared mental model (SMM) enhances performance toward a common goal by allowing effective collaboration through promoting the sharing of knowledge and skills among interprofessional team members. Therefore, this study developed a web-based educational program for LTCF interprofessional practitioners based on an SMM. We followed a network-based instructional system design that consists of analysis, design, development, implementation, and evaluation for developing the program. A total of 54 participants completed the educational program in four LTCFs in South Korea. A significant improvement was identified in communication knowledge, communication confidence, role recognition, transactive memory system, and team effectiveness in the experimental group. The results show that the program improved the emergency management process and reliability among interprofessional practitioners, positively impacting interprofessional collaboration and ensuring the safety of patients during emergencies in LTCFs.

## 1. Introduction

Interprofessional training aims to improve collaboration between the members of an interprofessional team so that emergencies such as falls, airway obstructions, and infections in long-term care facilities (LTCFs) can be managed in a successful way [1]. Collaboration between interprofessional team members has been shown to improve the quality of care provided to LTCF residents [2] and to have a positive effect on patient safety and health outcomes by reducing the frequency of adverse events [3]. In contrast, poor collaboration has been reported to adversely affect patient safety because it leads to prolonged hospital stays due to adverse events [4].

A systematized process, such as the sharing of knowledge, skills, roles, and goals that helps convert individual knowledge into team knowledge, is needed to improve collaboration [5]. To extend knowledge from the individual level to the team level, Cannon-Bowers et al. [5] proposed the shared mental model (SMM). The SMM allows team members to recognize and understand each other’s different areas of knowledge and roles, as well as to predict other team members’ behaviors, enabling effective collaboration [6]. Systematized knowledge at the team level enhances performance toward the shared goal of, for example, patient safety [7].

However, LTCFs struggle with limited resources and staffing [8]; hence, it is challenging in such an environment to provide sufficient practical training for improving collaboration. In order for the gap between environmental limitations and the demand to enhance practitioner competency in LTCFs to be reduced, web-based education has been considered an appropriate and effective strategy [9]. Moreover, web-based education is an alternative solution when face-to-face education is challenging in situations such as the COVID-19 pandemic, and it can improve the effectiveness of education through repeated and continuous learning [10]. Accordingly, this study developed and evaluated a web-based educational program based on an SMM design for LTCF interprofessional practitioners.

### Adapting SMM to This Study

The SMM is multidimensional and consists of four domains: “equipment,” “tasks,” “team interactions,” and “team members” [5,11]. In equipment, technical aspects and equipment are coordinated by sharing knowledge on equipment functions, operating procedures, equipment limitations, and the possibility of equipment failure [5]. Under task, task processes, strategies, scenarios, task results, work environment constraints, and relationships between tasks are included for predicting how a task will be performed [5]. The team interaction domain refers to team members sharing knowledge about their roles, responsibilities, flow of information, sources, interaction patterns, communication channels, and interdependence of roles [5]. In the final domain, team members, as well as knowledge and skills concerning team members’ characteristics, such as attitudes and preferences, are shared [5]. Cannon-Bowers and Salas [6] found that sharing the four domains of SMM with team members results in better task performance, team processes, and motivational outcomes.

**Figure 1:**
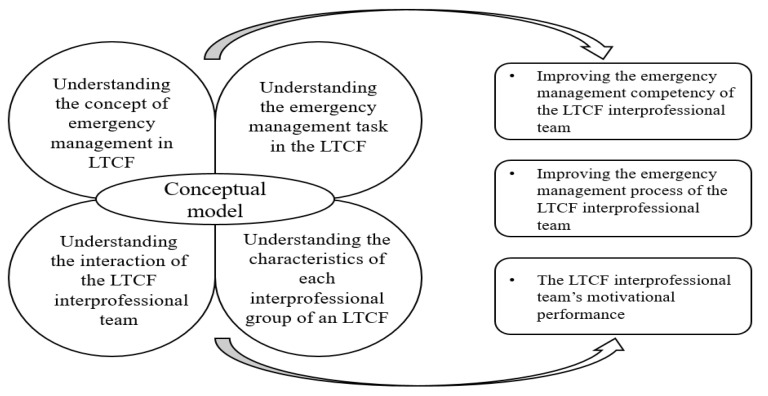
Conceptual model adapting the shared mental model; LTCF = long-term care facility.

The four multidimensional mental domains developed by Cannon-Bowers et al. [5] and the results of the SMM presented by Cannon-Bowers and Salas [6] were used to construct the conceptual framework of this study. The equipment domain of the SMM for emergency management in LTCFs is conceptualized as “understanding the concept of emergency management in LTCF,” which corresponds to the necessary procedures and skills required to manage emergencies. The SMM’s task domain was conceptualized in this study as an “emergency management task in the LTCF,” which corresponds to the procedures and strategies for emergency management, while the “team interaction” domain of the SMM was conceptualized as “understanding the interactions of the LTCF interprofessional team.” Finally, the SMM’s team members domain was conceptualized in this study as “understanding the characteristics of each member in LTCF interprofessional groups.” The results of the SMM include “improving the emergency management competency of the LTCF interprofessional team,” “improving the emergency management process of the LTCF interprofessional team,” and “improving the LTCF interprofessional team’s motivational performance.” An educational program for emergency management in LTCFs was developed on the basis of this conceptualized model (Figure 1).

## 2. Methods

**Figure 2:**
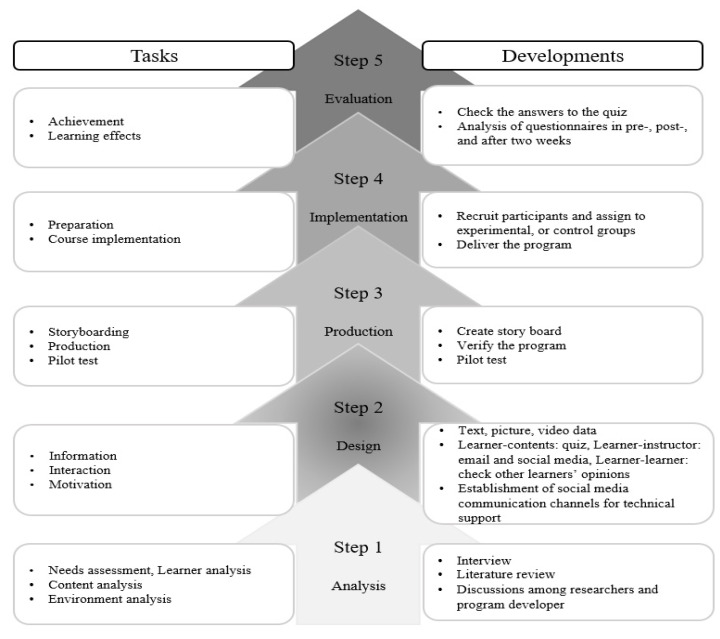
Study process of web-based educational program.

This study’s educational program was developed using the Network-Based Instructional System Design (NBISD) model [12], which is widely used in developing web-based educational programs [13]. The NBISD consists of five stages: analysis, design, production, implementation, and evaluation. Figure 2 shows the NBISD-based procedure used in this study.

### 2.1. Step 1. Analysis

The purpose of Step 1 was to assess the educational needs and to compose the educational content. For a learner needs analyses, this study identified the status of emergency management in four LTCFs and the knowledge and skills required for emergency management education through interviews with 32 LTCF practitioners. In addition, the scope of emergency management education for LTCFs was determined through a literature review. The educational contents consisted of organizing the existing literature on emergency management methods by focusing on frequent emergencies in LTCFs. A program developer was hired for environmental analysis, and discussions between researchers and the program developer were conducted to technically establish a web-based educational program.

### 2.2. Step 2. Design

The purpose of Step 2 was to construct educational content and teaching methods. In this step, the educational goals, contents, methods and strategies, environment, and evaluation were specified through the Consensus-Oriented Decision-Making (CODM) process and a content validity index (CVI) with an expert group for the areas of information design, text, pictures, and video data. Next, learners were asked to interact with each other while solving a quiz to check their understanding of the content on the basis of their interactions. The program was designed to allow learners to react to and to check each other’s opinions and answers, using email and social media as channels for interacting with other learners and instructors. Strategies for motivation, such as primary education, were established, and a communication channel with researchers through social media was also designed to provide technical support if needed.

### 2.3. Step 3. Production

Step 3 aimed to implement the learning content and system. The learning content was designed using various types of media for user interactions in learning procedures. Since the learning system focused on allowing users to study the contents at their desired times and locations, the learning content and system were developed in web-based environments. The first author (Y.R.) created a storyboard, after which the first and second authors (D.Y.) built a web browser, server, and database and conducted a pilot test to check for program operation errors.

### 2.4. Step 4. Implementation

The purpose of Step 4 was to enable learners to use the developed educational program. In this step, participants were recruited, and the educational program was conducted.

#### 2.4.1. Participants

**Figure 3:**
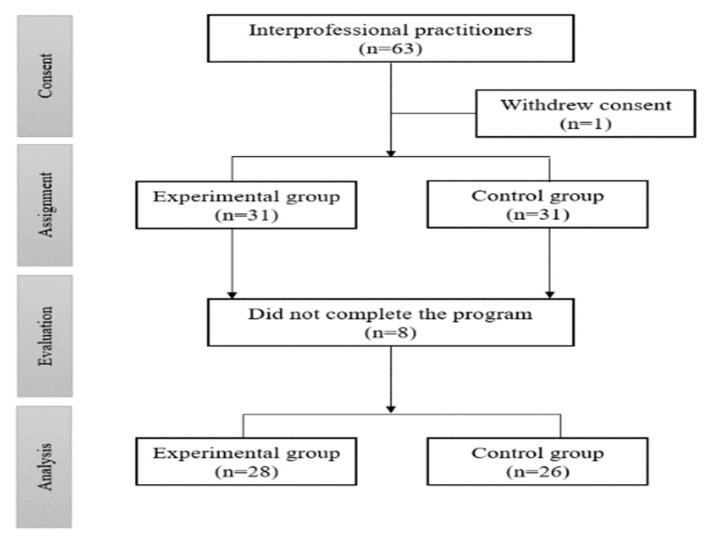
Process of the participants selection.

Figure 3 presents the process of participant selection used in this study. A total of 63 people participated, 54 of whom completed the educational program in four LTCFs in South Korea. The inclusion criteria for this study were as follows: (1) more than one year of work experience in an LTCF, and (2) currently working at an LTCF. G*Power 3.1.9.4 (Heinrich-Heine-Universität, Düsseldorf, Germany) was used to calculate the sample size. When the power was 0.8, the significance level was 0.05, and the effect size was 0.80 [14], on the basis of which both experimental and control groups of this study were confirmed to have proper sample size. Of the 54 participants in this study, 28 were in the experimental group and 26 in the control group.

#### 2.4.2. Procedure

After developing the web-based educational program, the researchers guided the participants through telephone interviews with the head of the facility and distributed by mail the written instructions on how to use the educational program. Due to COVID-19 restrictions, visiting each LTCF proved to be difficult. Therefore, the details regarding the educational program manual were provided over the phone and through the written document. The manual for using the educational program included how to access the web program, a table of contents for the program, how to input answers, and other related information. 

#### 2.4.3. Outcome Measures

The Korean version of “communication confidence,” “communication knowledge,” “role perception,” “transactive memory system,” and “team outcomes” were used to measure the learning outcomes. 

Communication confidence was measured on a scale of 0 to 10, with higher scores indicating higher confidence [15]. Cronbach’s α was 0.87 in this study. Communication knowledge was developed by the research team. To measure the item, we gave participants an example of an emergency and asked them what information should be delivered to other practitioners. This measurement tool contains four items that measure information about emergencies, the patient’s background, assessments, and recommendations. Two points were awarded for two or more correct information items, one point was awarded for one correct information item, and zero points were awarded for the wrong answer or failure to deliver the information. Role perception was also developed by the research team. The participants were asked to describe in writing their and other practitioners’ roles in managing emergencies and emergency prevention. For the four role perception questions, zero points were given if zero or one item was correct, one point was given if two items were correct, and two points were given if three or more items were correct. Cronbach’s α was 0.68 in this study. Transactive memory system was measured with 15 items on expertise, reliability, and task coordination scored on a Likert 5-point scale, with higher scores indicating a higher transactive memory system [16]. Cronbach’s α was 0.93 in this study. Team outcomes included team performance, commitment, and satisfaction; Cronbach’s α was 0.86 in this study. This tool also used a 5-point Likert scale, with higher scores indicating higher team outcomes [17].

### 2.5. Step 5. Evaluation

The purpose of Step 5 was to identify learning achievement. This study confirmed the results of the educational program by conducting an experiment through pre- and post-program surveys of the non-equivalence control group. The experimental group completed a web-based educational program based on the SMM, and the control group used an emergency management educational program that focused on knowledge (Table 1). The survey was performed three times: before, immediately after, and two weeks after using the web-based educational program. 

### 2.6. Data Analysis

SPSS version 25.0 was used to analyze the data. Descriptive statistics, a two-sample *t*-test, and repeated measures ANOVA were performed to confirm the differences between the before, immediately after, and two weeks after time points. Post hoc tests were performed using Bonferroni correction. All analyses were validated at a significance level of 0.05.

### 2.7. Ethical Considerations

This study was conducted after obtaining the IRB approval of the researcher’s institution (KUIRB-2019-0174-01). After explaining to the participants the purpose of the study, the contents of the research, and that their participation was voluntary and they could withdraw from the study at any time, we obtained written consent.

## 3. Results

### 3.1. Development of the Web-Based LTCF Emergency Management Educational Program

Table 2 shows the educational goals, environment, contents, methods and strategies, and evaluation of the developed educational program. Participants learned about the knowledge and skills necessary for emergency management, team interaction, and the role of team members according to the conceptual framework of the SMM.

**Figure 4:**
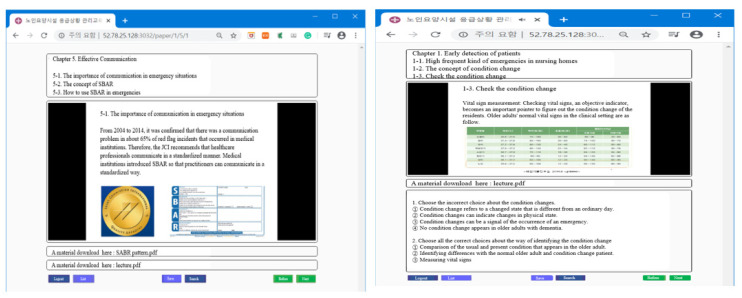
Example illustrations of web-based emergency educational program.

The web-based educational program developed consists of five chapters. Chapter 1 provides an overview of emergencies and early patient detection and includes the frequency of emergencies in LTCFs, concept of condition change, checking for condition change, professional knowledge, and the skills of interprofessional practitioners. Chapter 2 elaborates on minimizing patient injuries by providing definitions of airway obstructions, falls, and infections, while including information on patient assessment, first aid, and the role(s) of practitioners in emergencies. Chapter 3 focuses on the preparation of patient transport to the emergency department (ED) describing the patient information needed for treatment in the ED and the first aid that should be provided in the facility before transfer to an ED. Chapter 4 discusses the prevention of emergencies through identifying residents’ underlying diseases upon admission to LTCFs for determining those at high risk of experiencing emergencies, while depicting environmental management and the role of practitioners in preventing emergencies. Finally, Chapter 5 explains efficient communication between practitioners, the importance of communication, and the concept of the situation–background–assessment–recommendation (SBAR) framework and how to use it. Figure 4 shows the screen that the participants were able to view.

### 3.2. The Effects of the Web-Based LTCF Emergency Management Educational Program

The experimental and control groups were identified as homogenous groups in the homogeneity test for sociodemographic characteristics and dependent variables (Table 3). 

The results of the application of the educational program are presented in Table 4. 

Compared to the control group, the experimental group had significantly higher scores in communication confidence (t = 6.14, *p* = <0.001) after education, communication knowledge (t = 5.30, *p* = <0.001), role recognition (t = 3.29, *p* = 0.002), transactive memory system (t = 2.72, *p* = 0.009), and team outcomes (t = 3.21, *p* = 0.002). A repeated ANOVA showed that, for communication confidence (F = 19.82, *p* = <0.001), communication knowledge (F = 12.11, *p* < 0.001), role recognition (F = 20.06, *p* = <0.001), transactive memory system (F = 3.99, *p* = 0.021), and team outcomes (F = 8.69, *p* = <0.001), there were significant differences between the time points before, immediately after, and after two weeks of using the educational program. As a result of the post hoc analysis of the differences between the experimental group’s scores at the three time points, communication confidence (*p* = <0.001), communication knowledge (*p* = <0.001), role recognition (*p* = <0.001), transactive memory system (*p*= 0.016), and team outcomes (*p* = 0.023) were shown to have improved significantly after education.

## 4. Discussion

The emergency management capabilities of practitioners are being recognized as important because of the complex conditions of LTCF residents [18,19]. This study’s educational program contributes significantly to the learning of content for improving team collaboration ability during emergency management in LTCFs when compared to the existing education focused on knowledge and practice related to emergencies. Team collaboration is a form of interaction in which at least two members voluntarily participate in shared decision making toward a common goal [20]. Participants provided positive responses in the survey after using the educational program focusing on team collaboration improvement. 

Participants who learned through our educational program positively evaluated the comprehensive and practical contents of emergency management, such as early detection by checking a patient’s condition changes, preparations to transfer a patient to the ED, and how to prevent daily emergencies in contrast to the existing education focused on first aid. As LTCFs must cope with a high risk of emergencies regarding their residents’ conditions, practitioners who care for residents should be provided with training to develop their first aid skills and the ability to detect condition changes early to prevent emergencies [21]. Condition changes refer to changes in a resident’s health status that may appear before an emergency occurs, for example, a change in vital signs. Emergency management involves identifying condition changes through considering the underlying disease and frailty of the older resident [22]. Early detection of condition changes in residents by practitioners can prevent emergencies that may threaten residents’ safety.

Many residents have been transported to the ED from an LTCF. An average of more than 2 million residents in LTCFs are transferred to the ED for treatment each year in the United States [23], while the number of transfers to the ED was 1.37 times per resident in a Swiss nursing home [24]. Meanwhile, concerns have been raised about the safety of transferring residents. Unroe et al. [25] asserted that early patient detection, early intervention, and improved LTCF resources are needed to prevent unnecessary transfers to EDs. Accurate and sufficient patient information such as medical history, emergency onset time, and medications should be provided to physicians and nurses in the ED by LTCF practitioners. Hence, our educational program content included resident information required by ED staff for treatment. The program content also included first aid measures that LTCF practitioners should perform before transferring residents to the ED. 

Participants also stated that it was good to learn how to communicate in an emergency. Communication is one of the biggest challenges when people from different professions work together to achieve the same goal; however, standardized communication tools such as the SBAR tool can help close communication gaps among interprofessional team members [26]. Communication tools help the receiver understand what the sender of information is trying to convey, even in an emergency. Furthermore, using communication tools improves confidence and clinical performance and promotes smooth communication within a team [27]. We used the SBAR framework as a communication tool to improve communication among practitioners in LTCFs. This study also guides team members to recognize their roles and allows them to share their opinions with other team members [28].

Many LTCFs have staff and resource shortages, particularly during situations that threaten residents’ safety, such as the current COVID-19 pandemic, and their limited staff is overburdened with work [29]. Web-based education programs are a good solution and can be used in such situations. Molinari et al. [30] found that web-based education is an acceptable method for improving learners’ confidence. Our educational program was meaningful in that it was a web-based program that the learner could access at their desired times and locations. The program used lectures, case studies, and media data for providing educational content. This enabled learning and aided the participants to overcome the limitations of time and space, which is the most significant advantage of web-based education [31]. In addition, an asynchronous interaction element was provided for communication between the learners.

This study developed a practical education program based on the theoretical SMM. Significantly, the study supported the findings of previous research [7] by demonstrating improvement in team collaboration in the field of emergency management in LTCFs. In addition, the current approach is valuable because it provides comprehensive emergency management education, including training for emergency management and for improving interprofessionals’ ability to work in teams. This is consistent with previous work [32], which indicated that education that reflects the characteristics of a team is essential for improving the ability to collaborate.

This study shows that interprofessional practitioners can improve their collaboration ability by receiving education in role exploration and sharing and on communication for emergency management in the field. Future policy should encourage continuing education by developing and distributing programs in various subjects so that LTCF practitioners can develop emergency management collaboration skills on an ongoing basis. Moreover, future research should help develop educational programs by exploring the obstacles to web-based interprofessional education.

This study has several limitations. First, physicians were excluded from this study because, as visiting doctors and not LTCF resident doctors, they were not directly involved in emergencies [33]. Many modifications may be necessary if the contents of this educational program are to be applied in a facility where a physician resides. Moreover, classifying learning difficulty was not included in the program. Finally, a single program was applied to interprofessional practitioners with various educational backgrounds. To compensate for that, we used more accessible terms rather than medical terms for educational content. In addition, we used various pictures and video materials to match the education level of practitioners unfamiliar with medical terms and devices. An indirect form of cooperative learning was also implemented in which one participant checked the answers of other practitioners, who then got a chance to correct or supplement their responses. An emergency cannot be managed with one practitioner’s knowledge and skills; hence, a method for collaboration was suggested in this study for sharing the knowledge of other practitioners. Future studies could examine the exchange of ideas between learners in real time. We also recommend that future studies should conduct long-term follow-ups of more than two weeks to confirm the program’s long-term effects.

## 5. Conclusions

This study was conducted to develop and provide a web-based emergency management educational program based on an SMM design and to identify its effectiveness. The results of adapting the SMM showed that it improved interprofessional collaborations and could be applied even in emergency situations. The program provided a systematic curriculum to improve perceptions of increasing team collaboration during emergencies. This web-based program can be used as a continuous education program for practitioners in LTCFs, where residents’ safety demands are increasing. The contents of this education program can be modified and supplemented accordingly to fit different facilities, as long as they have a similar educational goal to this program. In this study, the educational content focused on airway obstructions, falls, and infections, as these are the most frequent emergency cases in LTCFs. Further studies are required to expand the types of emergency cases included in educational programs.

## Figures and Tables

**Table 1:** Experimental design.

	Before	Treatment	Immediately after	Two Weeks after
Experimental group	E_1_	X_1_	E_2_	E_3_
Control group	C_1_	X_2_	C_2_	C_3_

E_1_, C_1_: general characteristics, communication confidence, communication knowledge, role perception, transactive memory system, team outcomes; X_1_: web-based emergency management education program adapts a shared mental model for interprofessional practitioners; X_2_: web-based emergency management education program centered on knowledge transfer for interprofessional practitioners; E_2_ C_2_: communication confidence, communication knowledge, role perception, transactive memory system, team outcomes, satisfaction of educational program; E_3_ C_3_: communication confidence, communication knowledge, role perception, transactive memory system, team outcomes.

**Table 2:** An education curriculum for long-term care facility emergency management toward interprofessional teamwork.

Domain	Curriculum
**Goals**	Learners can explain how to identify the condition change(s) of residents in regard to airway obstructions, falls, and infections Learners can explain how to minimize the physical damage caused by airway obstructions, falls, and infections Learners can explain how to prevent an emergency situation due to airway obstructions, falls, and infections Learners can explain the key information needs for transferring a resident to an emergency department Learners can explain their own and other practitioners’ roles in managing emergencies Learners can use a communication tool when managing emergencies
**Environment**	Minimize shortcomings of on-site education (time and space limitations) Repeated and continuous education program Embrace all interprofessional practitioners linked with emergencies
**Contents**	Types and definitions of frequent emergencies in long-term care facilities Health characteristics of residents in long-term care facilities Concept of condition change and methods of identifying one Assessment for early detection First aid to minimize physical damage Identifying high-risk groups and their daily management Important information to deliver when transferring a patient to an emergency department Assignment of roles for practitioners Emergency management knowledge and skills of interprofessional team members Knowledge on the use of the SBAR framework
**Methods and strategies**	Present emergency patient case Provide a system and learning unit through problem-based learning Set goals for emergency management Provide learning materials and video data Provide quizzes Provide materials Organize learning contents by chapter
**Evaluation**	Communication knowledge Communication confidence Role recognition Transactive memory system Team effectiveness

**Table 3:** General characteristics of the participants and homogeneity test between experimental and control groups.

Variable	Category	Exp. (*n* = 28)	Cont. (*n* = 26)	*X*^2^ or t	*p*
		*n* (%) or M ± SD			
Age (years)	Average	48.14 ± 11.07	52.15 ± 10.27	−1.38	0.174
	≤30	4 (14.3)	3 (11.5)		
	31–40	3 (10.7)	1 (3.8)		
	41–50	8 (28.6)	5 (19.2)		
	51–60	10 (35.7)	10 (38.5)		
	≥61	3 (10.7)	7 (26.9)		
Gender	Male	2 (7.1)	2 (7.7)	0.06	1.000
	Female	26 (92.9)	24 (92.3)		
Education level	High school	7 (25.0)	7 (26.9)	5.54	0.136
	Associate degree	9 (32.1)	5 (19.2)		
	Bachelor	7 (25.0)	13 (50.0)		
	≥Master	5 (17.9)	1 (3.8)		
Job	Registered nurse	13 (46.4)	10 (38.5)	0.61	0.937
	Physical therapist	3 (10.7)	4 (15.4)		
	Social worker	4 (14.3)	4 (15.4)		
	Care worker	8 (28.6)	8 (30.8)		
Total work experience (years)		11.04 ± 6.25	12.19 ± 8.63	−0.57	0.573
Work experience in LTCFs (years)		5.50 ± 3.93	4.58 ± 2.64	1.01	0.320
Pre-scores of outcome variables					
Communication confidence		29.46 ± 3.92	29.65 ± 2.93	−0.20	0.842
Communication knowledge		2.18 ± 1.22	2.19 ± 1.23	−0.04	0.967
Role recognition		2.82 ± 2.18	2.96 ± 2.14	−0.24	0.813
Transactive memory system		55.50 ± 7.89	55.88 ± 6.15	−0.20	0.843
Team outcomes		20.29 ± 3.25	20.15 ± 2.92	0.16	0.876

Exp = experimental group, Cont = control group, M = mean, SD = standard deviation, LTCF = long-term care facility.

**Table 4:** Comparison of the scores between groups.

Variables (Range)	Time	Exp. (*n* = 28)	Cont. (*n* = 26)	T (*p*)	Source	F (*p*)	Bonferroni
		M ± SD					
Communication confidence (0–40)	Pretest	29.46 ± 3.92	29.65 ± 2.93	−0.20 (0.842)	group	17.75 (<0.001)	A < B, C
	Posttest	34.29 ± 3.45	29.35 ± 2.30	6.14 (<0.001)	time	19.82 (<0.001)	
	After 2 weeks	34.21 ± 3.37	29.81 ± 3.10	4.99 (<0.001)	group × time	21.22 (<0.001)	
Communication knowledge (0–8)	Pretest	2.18 ± 1.22	2.19 ± 1.23	−0.04 (0.967)	group	21.82 (<0.001)	A < B, C
	Posttest	4.25 ± 2.10	1.92 ± 0.80	5.30 (<0.001)	time	12.11 (<0.001)	
	After 2 weeks	4.57 ± 2.56	2.39 ± 1.20	4.02 (<0.001)	group × time	11.88 (<0.001)	
Role recognition (0–8)	Pretest	2.82 ± 2.18	2.96 ± 2.14	−0.24 (0.813)	group	5.41 (0.024)	A < B, C
	Posttest	5.39 ± 1.89	3.62 ± 2.08	3.29 (0.002)	time	20.06 (<0.001)	
	After 2 weeks	5.07 ± 2.04	3.54 ± 1.90	2.85 (0.006)	group × time	7.07 (0.001)	
Transactive memory system (0–70)	Pretest	55.50 ± 7.89	55.88 ± 6.15	−0.20 (0.843)	group	3.72 (0.059)	A < B, C
	Posttest	60.14 ± 6.39	55.88 ± 4.96	2.72 (0.009)	time	3.99 (0.021)	
	After 2 weeks	60.04 ± 6.69	56.35 ± 5.54	2.20 (0.032)	group × time	3.29 (0.041)	
Team outcomes (0–25)	Pretest	20.29 ± 3.25	20.15 ± 2.92	0.16 (0.876)	group	5.10 (0.028)	A < B, C
	Posttest	22.21 ± 2.36	20.15 ± 2.34	3.21 (0.002)	time	8.69 (<0.001)	
	After 2 weeks	22.50 ± 2.17	20.46 ± 2.73	3.05 (0.004)	group × time	6.13 (0.003)	

Exp = experimental group, Cont = control group, A = Pretest, B = Posttest, C = After 2 weeks.

## Data Availability

Data that support the findings of the study are available upon reasonable request from the corresponding author.
